# Exercise-induced anaphylaxis unrelated to food ingestion and with hyperleukotrieneuria during challenge testing

**DOI:** 10.1186/s13223-021-00593-8

**Published:** 2021-09-08

**Authors:** Chikako Motomura, Koji Ide, Terufumi Shimoda, Hiroshi Odajima

**Affiliations:** 1 Department of Pediatrics, National Hospital Organization Fukuoka National Hospital, 4-39-1 Yakatabaru, Minamiku, Fukuoka city, Fukuoka 811-1394 Japan; 2 Ide Kid’s Allergy Clinic, 3-32-19 Yokote, Minamiku, Fukuoka, 811-1311 Japan; 3 Department of Clinical Research, National Hospital Organization Fukuoka National Hospital, 4-39-1 Yakatabaru, Minamiku, Fukuoka, 811-1394 Japan

**Keywords:** Exercise-induced anaphylaxis, Urinary leukotriene E4, Cold-induced anaphylaxis, Pre-anaphylaxis stage, Mast cell activation

## Abstract

**Background:**

Exercise-induced anaphylaxis (EIA) is a rare and potentially life-threatening disorder that can develop independently without food ingestion. Cold drinks can also trigger symptoms in some patients with cold-induced anaphylaxis. We present a case of a patient with EIA that was diagnosed on the basis of positive exercise loading test with hyperleukotrieneuria.

**Case presentation:**

A 12-year-old girl presented with acute flushing, cyanosis, swollen eyelids, and dyspnea after an endurance run in winter or swimming in a cold-water pool. She also developed dyspnea after having a cold drink. She had no history of food allergies, atopy, or asthma. No association was noted between anaphylaxis and food intake in her history. On the first day, she ingested 200 mL of 5 °C cold water in 30 s, which did not trigger symptomatic responses, but her urinary leukotriene E4 (LTE4) level increased (pre-challenge test: 295 pg/mg-creatinine (cr), post-challenge test: 400 pg/mg-cr). On the second day, she underwent the exercise loading test according to the Bruce protocol by using an ergometer to increase the power of exercise every 2 min. She had been fasting for > 15 h and did not have breakfast. Just after the exercise loading test, the plasma adrenaline and noradrenaline increased. At 15 min after the exercise loading test, her plasma adrenaline and histamine (pre-challenge test: 0.7 ng/mL, 15 min post-challenge test: 81 ng/mL) rose sharply with anaphylaxis symptoms accompanied by increasing urinary LTE4 (pre-challenge test: 579 pg/mg-cr, post-challenge test: 846 pg/mg-cr). After she was discharged, she was restricted from strenuous exercise especially in cold environments and prescribed an adrenaline autoinjector.

**Conclusion:**

Cold stimulation can become a co-effector of EIA. Measurements of urinary LTE4 levels during challenge testing are useful for diagnosing EIA and capture the pre-anaphylaxis stage.

## Background

Exercise-induced anaphylaxis (EIA) is a potentially life-threatening disorder that can develop independently or in combination with food ingestion as food-dependent EIA (FDEIA). EIA without food ingestion is rare; few studies have reported positive challenge test results with elevated objective marker levels in patients with EIA [1]. Cold drinks can also trigger symptoms in some patients with cold-induced anaphylaxis.

Conversely, mast cells may play a role in cysteinyl leukotriene (CysLT) generation during anaphylactic reactions [2]. Urinary leukotriene E4 (LTE4) is the most reliable analytic parameter for monitoring endogenous CysLT synthesis. We herein present a case of a patient with EIA that was diagnosed on the basis of positive exercise loading test with hyperleukotrieneuria.

## Case presentation

A 12-year-old girl presented with acute flushing, cyanosis, swollen eyelids, urticaria, and dyspnea experience after an endurance run in winter or swimming in a cold-water pool. She also developed dyspnea after having a cold drink. She had no history of food allergies, atopy, or asthma. No association was noted between anaphylaxis and food intake in her history.

Laboratory assessment during the asymptomatic period revealed the absence of peripheral eosinophilia (white blood cell count, 5150/μL with 2% eosinophils) and a total immunoglobulin (Ig) E level of 1255 IU/mL. Specific quantitative IgE testing with ImmunoCAP^®^ > 0.7 UA/mL for house dust, dermatophagoides, cedar pollen, and cat dander and negative for wheat, shrimp, and crab. The skin-prick test also was negative for wheat, shrimp, and crab. On the basis of the symptomatic episode, she underwent a 2-day challenge test to measure urinary LTE4 levels before and 3 h after the test. Urinary LTE4 was quantified by using a commercial enzyme immunoassay kit (Cayman) after purification by high-performance liquid chromatography.

**Fig. 1:**
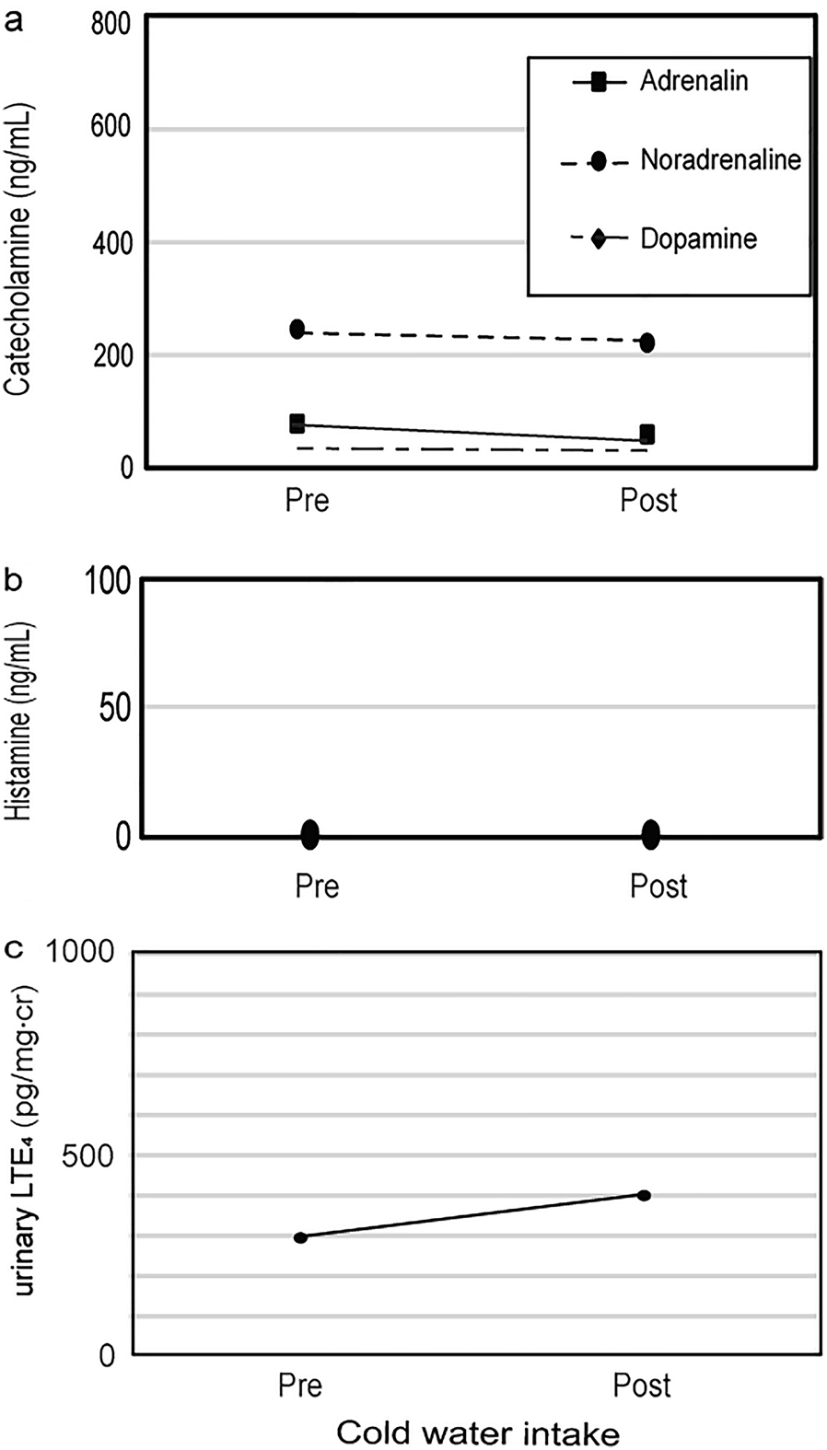
Changes in plasma catecholamine (**a**), histamine levels (**b**), and urinary LTE4 levels (**c**) by cold-drink challenge testing

**Fig. 2:**
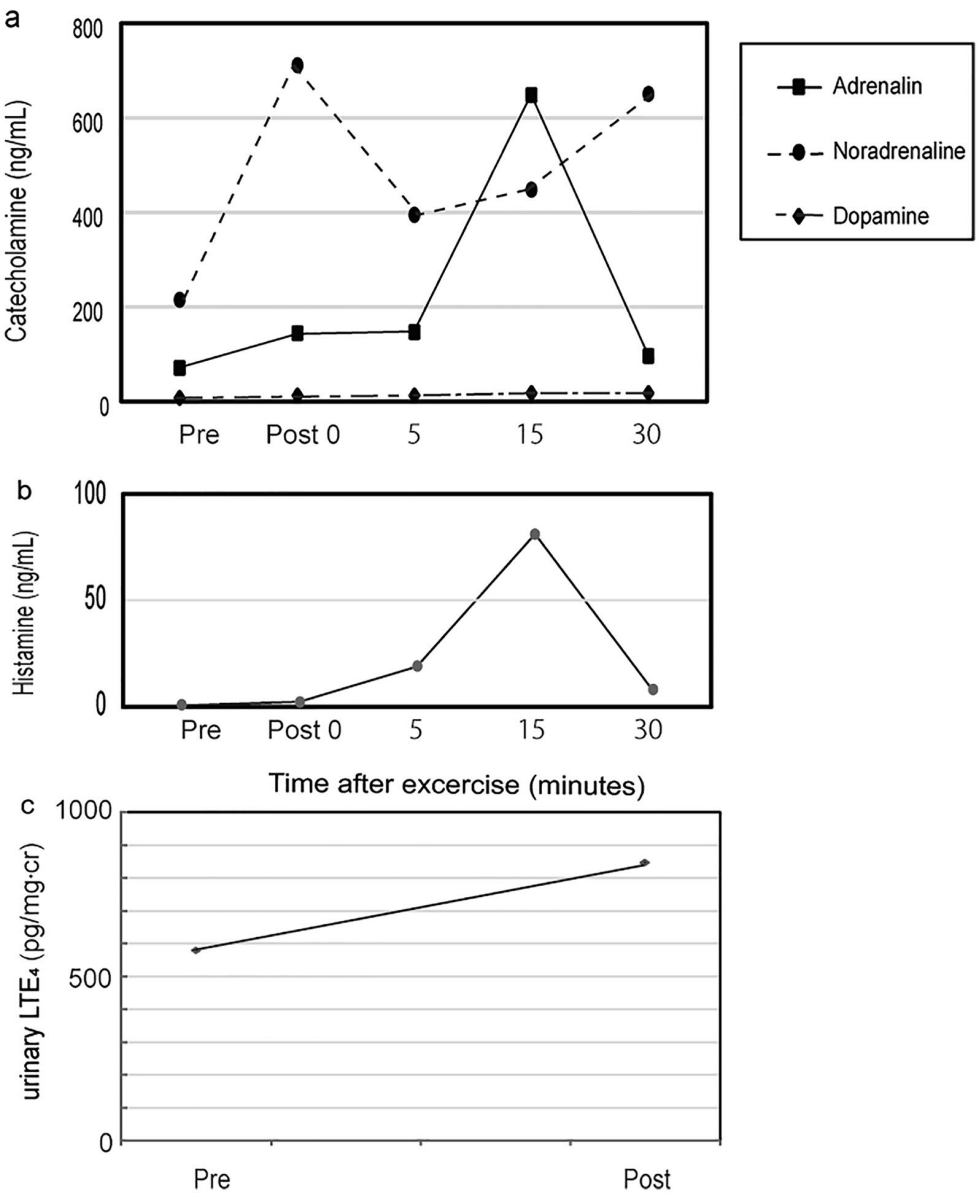
Changes in plasma catecholamine (**a**), histamine levels (**b**), and urinary LTE4 levels (**c**) by the exercise loading test

On the first day, she was asked to ingest 200 mL of cold water at a temperature of 5 °C in 30 s, which did not trigger any symptomatic responses. After the challenge test, the plasma catecholamine (adrenaline, noradrenaline, and dopamine) and histamine levels did not significantly alter (Fig. 1a and b), but her urinary LTE4 level increased (Fig. 1c, pre-challenge test 295 pg/mg-creatinine (cr), post-challenge test 400 pg/mg-cr). On the second day, she underwent the exercise loading test according to the Bruce protocol [3] by using an ergometer to increase the power of exercise every 2 min. She had been fasting for > 15 h and did not have breakfast. She developed acute flushing, cyanosis, swollen eyelids, dyspnea, and hypotension when the maximum loading reached 80 watts. Just after the exercise loading test, her plasma adrenaline and noradrenaline increased (Fig. 2a). At 15 min. after the exercise loading test, her plasma adrenaline and histamine (Fig. 2b, pre-challenge test: 0.7 ng/mL, 15 min post-challenge test: 81 ng/mL) rose sharply with anaphylaxis symptoms, such as cyanosis, dyspnea, and hypotension. Those symptoms were accompanied by increasing urinary LTE4 (Fig. 2c, pre-challenge test: 579 pg/mg-cr, post-challenge test: 846 pg/mg-cr).

The diagnosis of EIA had to be distinguished from systemic mastocytosis, exercise associated laryngoesophageal reflux, cardiovascular disorders, cholinergic urticarial, and hypoglycemia. The episode was confirmed to be EIA that was diagnosed on the basis of positive exercise loading test with hyperleukotrieneuria. Her serum tryptase level was 1.3 μg/L, within the normal range when she was asymptomatic.

After challenge testing, the patient was treated with anti-LT receptor antagonists. During next hospitalization, to improve her quality of life related to exercise, training by exercise prescription was conducted. Two weeks later, she increased the exercise intensity without anaphylaxis. After she was discharged, she was restricted from strenuous exercise especially in cold environments and prescribed an adrenaline autoinjector.

## Discussion and conclusions

In this case, anaphylaxis with hypotension developed after exercise without food intake and was accompanied by elevated levels of plasma histamine and urinary LTE4, both of which are clinical markers of mast cell activation [4]. Histamine is immediately released by mast cell degranulation that is activated by C3a and others. Plasma histamine levels were reported to be elevated in patients with FDEIA who had a positive challenge test [5]. Conversely, CysLT is derived from mast cell membranes within minutes, and urinary LTE4 levels increase by about tenfold from baseline after a positive challenge test in anaphylactic patients [2]. For other allergic and inflammatory disease, the urinary LTE4 increased in atopic patients after allergen inhalation and patients with eosinophilic vasculitis [6]. In both aspirin-intolerant asthma and aspirin-tolerant asthma patients, urinary LTE4 decreased after the sinus surgery. Recent studies have shown that the change in tryptase from baseline may be a more valuable parameter for diagnosing anaphylaxis [7]. We did not measure the change in tryptase during provocation testing in this case. Systemic mastocytosis was denied because the baseline tryptase was in the normal range.

The mechanism underlying EIA, especially when not caused by specific food exposure, has not been completely elucidated; however, several hypotheses have been proposed. First, reduced cellular pH in lactic acidosis due to supramaximal exercise was proposed as a way that could promote mast cell degradation and increase the propensity toward anaphylaxis [8]. Second, mild-to-severe exercise alters blood flow distribution, with a greater percentage of cardiac output going to active tissues such as skeletal muscle accompanied by a reduction in blood flow to the visceral organs, primarily the stomach and intestines. Functional heterogeneity has been demonstrated in human mast cells from different tissues. Therefore, the quantity and spectrum of mediators released may differ across different locations as well as under different situations [9]. These hypotheses warrant further investigation by comparing EIA patients with healthy controls.

Notably, urinary LTE4 levels reportedly did not increase after exercise in healthy children [10]. The urinary LTE4 level in our patient increased in both exercise loading and cold stimulation despite the negative result with cold-water intake; in the past, the patient had developed cold-induced anaphylaxis with urticaria while swimming in a pool. Generally, both mast cell and recruited basophils release histamine and other inflammatory mediators (prostaglandins, leukotrienes, and cytokines), leading to the activation of urticarial lesions. The same mechanism occurs systemically during cold-induced anaphylaxis. Interestingly, a study reported two patients with FDEIA who developed symptoms induced by physical exercise at cold temperatures [11]. However, the rate of anaphylactic reactions induced by cold stimulation in patients with EIA or FDEIA is unknown.

Our patient may have developed EIA. We found that plasma histamine levels transiently increased after exercise loading in this patient; however, this increase was not observed after the cold-water intake challenge test and was without symptoms. Stringent criteria in selecting subjects as well as performing repeated provocation tests might be needed to detect transiently increased histamine levels, even in FDEIA provocation tests [5]. Given that cold-water intake stimulation specifically to the throat mildly activates mast cells and basophils, we could knew the pre-anaphylaxis stage to measure the elevated urinary LTE4 level even without any symptoms present.

There have been some reports of successfully using anti-IgE biologics to treat EIA [1] and cold-induced anaphylaxis [12]. The mechanism by which non-specific IgE-dependent mast cell activation can be provoked by exercise, cold stimulation, or both in such cases is unclear. The established efficacy of anti-IgE biologics in the treatment of allergic asthma and allergic rhinitis putatively reflects the depletion of free IgE from serum and tissue, ultimately leading to reduced binding of IgE to its high-affinity surface receptor FcεRI. Because the occupancy of FcεRI by IgE determines the levels of surface FcεRI expression, this leads to a rapid depletion of both cell-bound IgE and surface FcεRI expression on blood basophils and a more gradual depletion of these proteins from skin and other organs’ mast cells [13].

We believe that cold stimulation can be a co-effector for EIA. Measurements of urinary LTE4 levels during challenge testing were useful for diagnosing EIA. The change in urinary LTE4 from baseline might be sufficiently sensitive to capture the pre-anaphylaxis stage at which provocation tests do not induce symptoms. In the future, conducting studies to elucidate the mechanism of non-specific IgE-mediated activation of mast cells, which can drive allergic reactions in patients with anaphylaxis induced by both exercise and cold stimulation, would be beneficial to further our understanding of these mechanisms and potentially improve management.

## Abbreviations

EIA: Exercise-induced anaphylaxis
FDEIA: Food-dependent EIA
CysLT: Cysteinyl leukotriene (CysLT
